# The Use of Alcohol in the Treatment of Nervous and Mental Diseases1Read at the Buffalo Academy of Medicine in discussion of the subject, “The use of alcohol in the treatment of diseases.”

**Published:** 1899-06

**Authors:** Sydney A. Dunham

**Affiliations:** Buffalo, N. Y.


### THE USE OF ALCOHOL IN THE TREATMENT OF NERVOUS AND MENTAL DISEASES.[1]

By SYDNEY A. DUNHAM, M. D., Buffalo, N. Y.

THE word alcohol in the treatment of disease means "whiskey or brandy," unless otherwise stated, according to Hare's Therapeutics, but it could be applied to any of the so-called alcoholic stimulants. By the judicious use of alcohol, in the treatment of acute disease, few have become addicted to its use. Through its indiscriminate use as a "pick-me-up" in disease of the nervous system, many have become habitués. It does not matter whether the physician treats the disease, or the patient treats himself, if alcohol is used our subject remains the same.

Alcohol for appetite, for sleeplessness, for that tired feeling, for extra work, does much harm and little good. Whenever it is used as a substitute for food and rest the effect is treacherous and deceptive. That work had better be left undone, rather than to depend upon the energy produced by a few extra drinks. It is better to live longer and have more time to work, than to do brilliant work for a short time and die of paresis caused by alcohol.

The following questions present themselves to us for our solution: Does alcohol do good in the treatment of nervous diseases? Does it do harm? Can it be replaced by other drugs? Those who most frequently abuse the use of alcohol are the neuropathics. Psychopathy, with its varied mental disturbances is the common result of the neuropaths—excessive use of alcohol. The false teaching of the use of alcohol years ago, as a safe remedy in the treatment of disease led to its use in moderation as a beverage and a remedy. But moderation has proven to be the forerunner of excess. With excess comes mental, moral and physical deterioration. Moderation in stimulants or narcotics is unknown to the unstable nervous system. The use of alcohol in the treatment of the following nervous diseases is not indicated but rather contraindicated:

*Diseases of the Brain.*—Cerebral congestion, hemorrhage, inflammation, embolism, thrombosis, tumors and abscesses.

*Diseases of the Spinal Cord.*—Spinal inflammations, e. g., meningitis, myelitis and poliomyelitis, sclerosis and paralysis.

*Diseases of the Peripheral [and Sympathetic Nerves.*—Neuritis neuralgia, spasms, cephalalgia, migraine.

1 Read at the Buffalo Academy of Medicine in discussion of the subject, "The use of alcohol in the treatment of diseases."

Alcoholic stimulants are contraindicated in epilepsy, hystero-epilepsy and syphilis of the nervous system. They are pernicious in their effect in neurasthenia, chorea and hysteria. Syphilitic gumma of the brain frequently leads to excessive use of alcoholics.

Professor H. C. Wood, of Philadelphia, in his Work on Therapeutics and Materia Medica, says: "In chronic neuralgia, in hypochondriasis, melancholia, temporary relief may be obtained by the use of stimulants, but the very relief obtained doubles the temptation, by the frequent use of alcohol and the habit of frequent stimulation, grows almost of necessity into drunkenness. For this reason I do not think the physician ever justified in prescribing alcohol for its narcotic stimulant effect in these cases."

Some nervous systems are very sensitive to alcohol as a narcotic, hence in those cases where it is used to relieve pain—dysmenorrhea and neuralgias in general—and when used to produce sleep, there is danger of its use leading to excess with consequent evils.

While it has little use in nervous diseases, it is less indicated in mental affections.

"Alcoholic stimulants should never be given to periodical lunatics without bearing in mind the danger of formation of the dipsomaniacs tendency. In the free intervals of periodical mania alcoholic beverages are not borne well, a moderate indulgence leading to disproportionate disturbances of consciousness and will power."—Spitzka's Manual of Insanity.

As a toxic agent to the nervous system in producing insanity, it stands next to syphilis. In mental diseases there is no occasion for the use of alcohol. Any diseases caused by the use of alcoholic stimulants certainly could not be cured by continuing its use.

Chapin's Compendium of Insanity (physician to Pennsylvania Hospital for the Insane) says: "In melancholia the prolonged administration of hypnotic drugs has the effect of superadding to the existing mental disorder a new pathological state and a general sense disturbance."

In our State Hospitals they depend largely upon the nutritious food rather than drugs. There is no place for its use in imbecility, nor idiocy, nor mania, nor melancholia, nor paresis, nor dementia, nor any of the psychoses. Its contraindications in mental diseases are more definitely set forth by Hugo Hoppe, of Berlin. He mentions the following cases in which no alcohol should be given: First, chronic alcoholics which compose 50 per cent. of the insane in Berlin, in 1893; second, epileptic insane, which composed 8 to 10

per cent. of the insane; third, paretic dements, where alcohol is very injurious, about 15 per cent. of insane; fourth, idiots and imbeciles, about 13 per cent.; fifth, periodic insanities and maniacal patients, 5 per cent. In fact he believes in discontinuing the use of alcohol in insane asylums.

The most recent and valuable knowledge regarding alcohol and insanity is found in the ninth annual report of the State Commissions in Lunacy, by Dr. Ira Van Gieson, who has charge of the pathological institute for the scientific investigation of insanity. The ninth annual report of the commission in lunacy is the most valuable one ever printed and is an addition to any medical library, on account of its original information and ideas presented for the prevention of insanity. He says in this report that "alcohol begins its dissolution of the nervous system at the very highest centers of self-control and discrimination. The action of alcohol upon the nerve cells is to lessen its capacity to store energy, which they receive in the food supply from the blood-vessels. That alcohol in accordance with the law, that the highest and most precious portions of our brain are the last to become evolved and educated, are the most unstable and are also the most ready to undergo dissolution in the presence of any noxious stimulant." In speaking of preventive measures, he says: "Above all, the action of alcohol upon the nerve cell should be impressed upon the minds of growing children as soon as they are able to assimilate such knowledge."

Herein, then, lies the secret and the solution of the problem of why it is an individual cannot stop drinking when it is injuring him. His diseased imaginations, with weakened will-power, compel him to drink again. If the chief functions of a nerve cell are disturbed and perverted, such as coördination, transference, generating impulses (automatism) and other vital organs are dependent upon the nerve centers for energy and control, it is not strange that moderation leads to excess and excess to insanity, presenility and early death. The moderate drinker appears healthy, but there is often a gradual toxemia.

The use of alcoholic liquors in a general way with the degenerate, or those who have unstable nervous systems is always an abuse. With some it causes nervous instability, with others we find nervous instability predisposing excess in drink. In either case there is great responsibility upon the one who first suggested its use. Alcohol exceeds opium in its disastrous effects. The greatest danger from alcohol is not in the physician using it at the bedside as a medicine, but in his indifference and neglect to advise those in health or think themselves sick to abstain entirely.

The following statistics being so near home and many of the physicians being taxpayers may emphasize more than anything else why a physician should be so careful in prescribing alcohol in disease and allowing patients to use it so indiscriminately. In Buffalo in 1897, there was 10,319 arrests for drunkenness. Of this number 4,643 were sent to the Erie County Penitentiary. The ninth annual report of the State Commission in Lunacy, ending September 30, 1897, gives of 4,649, the original number of commissions for that year 534 from intemperance—458 men, 76 women. At the Matteawan Hospital for the criminal insane for 1897, out of 154 committed, 18 were for intemperance. The Craig Colony for epileptics for 1897, out of 253 inmates, alcoholism was traced to parents of 74 and to 26 as a direct cause of epilepsy. When we add to these reports the inmates of homes for feeble-minded children and deaf mutes produced by alcoholic drinks, we must admit there is no preventable disease with such disastrous results, not even tuberculosis or cancer. No wonder death and taxes are the most certain things in the world.

Alcohol on the nervous system lessens the resisting power by exhausting and paralysing the nerve centers. Syphilis is the chief cause of the chronic incurable diseases of the nervous system. Abuse of alcohol follows next as a degenerator of nerve tissue. We agree that the excesses in alcohol produce physical decay; why not admit that the first evil effects is through the nervous system by producing mental and moral perversions, and that Bright's disease is the effect of disturbance of trophic nerves produced by alcohol? Alcohol not only degenerates the healthy, but degenerates the degenerate and the degeneracy is handed down to posterity. Without alcohol, many an unstable nervous system would be able to preserve itself and to compete with the various activities of life, but by its use becomes helpless and a state care. If we are to prevent the increase in mental diseases and lessen the frequency of nervous trouble, alcohol must not only be dropped as a beverage, but used cautiously as a therapeutic agent. Alcohol is not indispensable in any disease. It can readily be replaced by other substances. Prudent physicians are cautious today about prescribing alcohol and do not when other remedies act as well and could be used instead.

Tell us in what diseases alcohol could be used as a prophylactic and curative agent and something else have not done as well?

When used indiscriminately and thoughtlessly in disease, it will do more harm than good. It is better to have died with disease than to have been saved by a drug which enslaves and produces habits which cannot be broken and thereby become a pauper and a dotard.

The "LaSalle," Chippewa Street.

